# Functional Properties of Poly(Trimethylene Terephthalate)-Block-Poly(Caprolactone) Based Nanocomposites Containing Graphene Oxide (GO) and Reduced Graphene Oxide (rGO)

**DOI:** 10.3390/nano9101459

**Published:** 2019-10-15

**Authors:** Sandra Paszkiewicz, Daria Pawlikowska, Magdalena Kurcz, Anna Szymczyk, Izabela Irska, Rafał Stanik, Maik Gude, Amelia Linares, Tiberio A. Ezquerra, Ludwika Lipińska, Michał Woluntarski, Agata Zubkiewicz, Elżbieta Piesowicz

**Affiliations:** 1 Institute of Material Science and Engineering, West Pomeranian University of Technology, Szczecin, Piastów av. 19, PL-70310 Szczecin, Poland; daria.pawlikowska@zut.edu.pl (D.P.); izabela.irska@zut.edu.pl (I.I.); senel@zut.edu.pl (E.P.); 2 Institute of Electronic Materials Technology, 133 Wólczyńska str., PL-01919 Warsaw, Poland; magdalena.kurcz@itme.edu.pl (M.K.); ludwika.lipinska@itme.edu.pl (L.L.); michal.woluntarski@itme.edu.pl (M.W.); 3 Institute of Physics, West Pomeranian University of Technology, Szczecin, Piastów Av. 48, PL-70311 Szczecin, Poland; Anna.szymczyk@zut.edu.pl (A.S.); Agata.zubkiewicz@zut.edu.pl (A.Z.); 4 Institute of Lightweight Engineering and Polymer Technology, Technische Universität Dresden, Holbeinstraße 3, 01307 Dresden, Germany; rafal.stanik@tu-dresden.de (R.S.); maik.gude@tu-dresden.de (M.G.); 5 Instituto de Estructura de la Materia, IEM-CSIC, Serrano 121, 28006 Madrid, Spain; alinares@iem.cfmac.csic.es (A.L.); t.ezquerra@csic.es (T.A.E.)

**Keywords:** block copolymers, graphene oxide, reduced graphene oxide, in situ polymerization, electrical conductivity, thermal conductivity, mechanical properties, morphology

## Abstract

This work reports a study on the influence of graphene oxide (GO) and reduced graphene oxide (rGO) on the functional properties of poly(trimethylene terephthalate)-block-poly(caprolactone) (PTT-*block*-PCL-T) (75/25 wt.%/wt.%) copolymer, obtained from dimethyl terephthalate (DMT), 1,3-biopropanediol and polycaprolactone diol (PCL) via in situ polymerization. The article presents, if and how the reduction of graphene oxide, in comparison to the non-reduced one, can affect morphological, thermal, electrical and mechanical properties. SEM examination confirms/reveals the homogeneous distribution of GO/rGO nanoplatelets in the PTT-*block*-PCL-T copolymer matrix. More than threefold increase in the value of the tensile modulus is achieved by the addition of 1.0 wt.% of GO and rGO. Moreover, the thermal conductivity and thermal stability of the GO and rGO-based nanocomposites are also improved. The differential scanning calorimetry (DSC) measurement indicates that the incorporation of GO and rGO has a remarkable impact on the crystallinity of the nanocomposites (an increase of crystallization temperature up to 58 °C for nanocomposite containing 1.0 wt.% of GO is observed). Therefore, the high performances of the PTT-*block*-PCL-T-based nanocomposites are mainly attributed to the uniform dispersion of nanoplatelets in the polymer matrix and strong interfacial interactions between components.

## 1. Introduction

The necessity of searching for renewable raw materials (feedstocks) for plastic production is well justified by decreasing fossil fuel resources, increasing crude oil prices, emission of greenhouse gasses in standard methods of plastic production and limited biodegradability of plastic materials [1,2,3]. These drawbacks make biocomposites more and more interesting for industrial applications. Biocomposites can be bio-based or biodegradable, or both. They contain at least one biomass materials such as corn, starch, cellulose, lignin, proteins, vegetables oil, etc. or biodegradable component (component that returns to nature) like polycaprolactone (PCL), poly(lactic acid) (PLA), poly(hydroxybutyrate) (PHB), poly(ethyl acrylate) (PEA), etc. [1,3,4,5,6]. Bio-based plastics are known over a century longer than petrochemical plastics. The first artificial bio-based thermoplastic polymer was celluloid, developed by Anselme Payen in 1838 [7]. Since then, many bio-based plastics have been tried out, but most of them have never been commercially exploited, due to the development of cheaper, synthetic polymers obtained from crude oil in the 1950s [4]. Currently, when the interest in biopolymers is growing, there is a need to produce biopolymers with good mechanical, thermal, barrier and other functional properties, which depend on the applications. One of the solutions to improve their properties is to use carbon derivatives (graphene oxide, graphene nanoplatelets, carbon nanotubes, etc.) as fillers. 

Graphene is a single layer of carbon atoms arranged in a honeycomb crystal structure that shows excellent properties such as high Young’s modulus ~1 TPa [8], intrinsic strength ~42 N/m, thermal conductivity ~4840–5300 W/(m·K) [9,10,11], electrical conductivity 7200 S/m [12] and good optical transparency ~97.7% [8]. Unfortunately, it is hard to produce a large amount of graphene and that makes it expensive. Graphene oxide (GO) and reduced graphene oxide (rGO) show similar properties (as fillers) and they are less expensive than graphene, furthermore they can be obtained in large amounts [8]. Graphene oxide is a synthetic compound, made for the first time by chemical treatments of graphite with potassium chlorate (KClO_3_) and fuming nitric acid (HNO_3_) by Brodie [13]. One can produce GO by the oxidative treatment of graphite it can be done via the Brodie [14], Staudenmeier [15], Hummers [16] or Tour [17] method. GO is some kind of electronically hybrid material that features both conducting π-states from sp^2^ carbon sites and a large energy gap (carrier transport gap) between the σ-states of its sp^3^-bonded carbons [18]. It can be transformed from an insulator to a semiconductor by chemical reduction [13,18,19] that tunes its band gap. Furthermore, GO exhibits good solubility and processability in water and in several organic solvents, which makes it easy handling. It can be used as reinforcement of polymer composites, because of its excellent gas-barrier properties, which result in a high aspect ratio [20] and high intrinsic mechanical properties [20,21,22]. Reduced graphene oxide (rGO) can be obtained by thermal [23], electrochemical [24,25] or chemical [26,27] reduction of graphene oxide. These methods help to remove the oxygen functionalities from the surface of GO and would result in varying graphene performances in terms of electronic, structural, physical and surface morphological properties [26]. Reduced graphene oxide has higher conductivity than GO [23,28] and its elastic modulus is similar to value predicted for pristine graphene [28]. Therefore, due to these exceptional characteristics graphene oxide and reduced graphene oxide are found to be promising candidates for improving functional properties of the wide range of polymer matrices [29], especially block copolymer matrices [30,31,32]. For instance, Wang et al. [30] investigated electrical and mechanical properties of styrene–butadiene–styrene tri-block copolymer (SBS) nanocomposites containing SBS-grafted graphene oxide (SBS-*g*-GO) nanofillers dispersed in the SBS matrix through a solution processing method. They showed that SBS molecules were homogeneously bonded onto the surface of the GO, leading to an improvement of the mechanical and electrical properties of SBS/SBS-*g*-GO composites due to the excellent interfacial adhesion and dispersion of SBS-*g*-GO in SBS. Moreover, in our previous studies based on poly(trimethylene terephthalate-*block*-poly(tetramethylene oxide) copolymer (PTT-*block*-PTMO) nanocomposites [31,32] we have shown how the addition of GO affected the two-phase structure, melt viscosity and mechanical properties of the polymer matrix and in addition, we compared its influence with other 2D-type nanofillers, i.e., graphene nanoplatelets (ANGSTRON Materials, Dayton, Ohio, USA) and modified organoclay (layered silicate based on montmorillonite, Nanofil 32, Süd-Chemie, Germany). However, to the best of our knowledge, there is no study dealing with the influence of GO and rGO on the properties of block copolymers partially based on renewable resources, like poly(trimethylene terephthalate)-*block*-poly(caprolactone) copolymers (PTT-*block*-PCL-T).

Synthesis details and properties of a series of PTT-*block*-PCL-T were described in our previous work [33]. It was found that the most promising system for obtaining polymer nanocomposites containing graphene derivatives is the PTT-*block*-PCL-T 75/25 wt.%/wt.%. In order to obtain copolymers with improved functional properties, like electrical and thermal conductivities a series of PTT-*block*-PCL-T nanocomposites with graphene oxide (GO) and reduced graphene oxide (rGO) as nanofiller were prepared. The composition and chemical structure of the prepared series of PTT-*block*-PCL-T based nanocomposites were confirmed by Fourier transform infrared spectroscopy (FTIR) [29]. The degree of GO and rGO distribution within the polymer matrix was observed by scanning electron microscopy (SEM). The morphology and crystallization behavior of the synthesized nanocomposites were analyzed with differential scanning calorimetry (DSC). Moreover, the tensile properties, electrical and thermal conductivity, thermal and thermo-oxidative stability and intrinsic viscosity were studied. 

## 2. Materials and Methods 

### 2.1. Materials

#### 2.1.1. Polymer Matrix

The following reagents were used in order to obtain PTT-*block*-PCL-T based nanocomposites: α,ω-telechelic polycaprolactone (PCL) diol (Polysciences Europe GmbH, Hirschberg, Germany) with the number molecular weight of 1250 g/mol, dried for 2 h under vacuum at 60 °C before use; Susterra^®^ 1,3-propanediol (bio-derived PDO, DuPont Tate and Lyle BioProducts, Loudon, TN, USA) purified by distillation before use; dimethyl terephthalate (DMT; Sigma-Aldrich, St. Louis, MO, USA); tetrabuthyl orthotitanate used as the catalyst (Sigma-Aldrich, St. Louis, MO, USA) and Irganox 1010 (Ciba Geigy, Basel, Switzerland) applied as a thermal stabilizer, used as received.

#### 2.1.2. Nanofillers: Graphene Oxide (GO) and Reduced Graphene Oxide (rGO)

Graphene oxide was prepared using natural graphite (Asbury #635, Detroit, MI, USA) as a starting material, by the modified Hummers method. The other reagents were sulphuric acid 96% (Chempur, Piekary Slaskie, Poland) 750 mL, sodium nitrate (Chempur, Piekary Slaskie, Poland) 16.5 g, potassium permanganate (Chempur, Piekary Slaskie, Poland) 90 g and hydrogen peroxide 30% (Chempur, Piekary Slaskie, Poland) 30 mL. In the beginning, sodium nitrate was added to sulphuric acid and mixed until dispersed completely, then graphite was added and stirring was being continued. The temperature was lowered to 5 °C. Then, potassium permanganate was added in a few portions, the reaction mixture was heated and maintained around 50 °C and stirred for several hours. Finally, the postreaction slurry was heated to up to 90 °C and diluted in cold water, and after that hydrogen peroxide was added. The purification process contains several sedimentation stages. Then, several washing cycles with deionized water were done by centrifugation. Next, the suspension of GO was exfoliated by the ultrasound probe (500 W, 30 min, ampl. 75%).

GO A635 sample was dried using the freeze-drying process. The second sample: rGO B635 was obtained by reduction, using a water solution of sodium hypophosphite. The reaction was conducted at 100 °C. After completion of the reduction process, the postreaction mixture was purified. The obtained product was dried using a freeze dryer to obtain rGO B635 in the form of powder.

**Figure 1:**
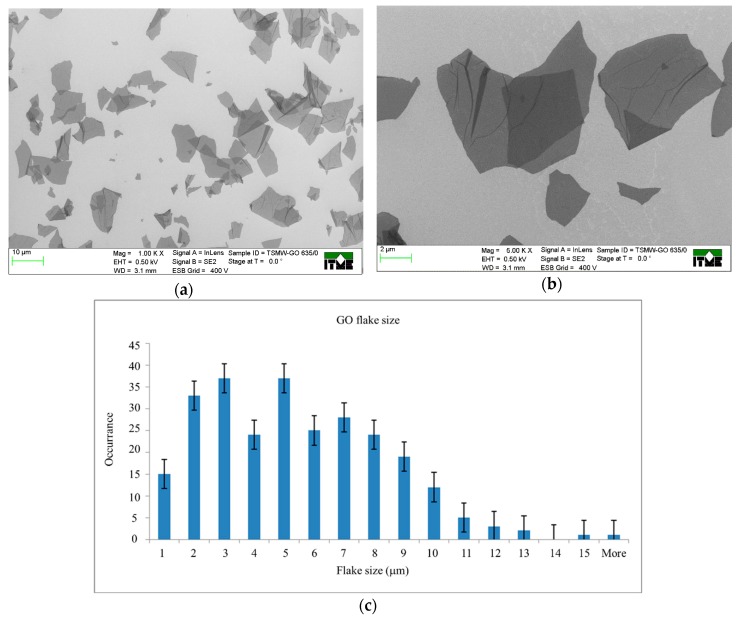
SEM images of GO (A 635) at magnifications: (**a**) 1.00 K × and (**b**) 5.00 K ×; (**c**) The size distributions of GO flakes (population of over 260 flakes was taken under consideration).

The SEM analysis shows flakes morphology. For imaging of GO, the diluted suspension is drop-cast on conducting silicon surface. The majority of flakes have a linear size of around 5 µm (Figure 1). Observed wrinkles are typical for thin, gentle flakes and they are caused by the way of preparing a sample for SEM examination. 

**Figure 2:**
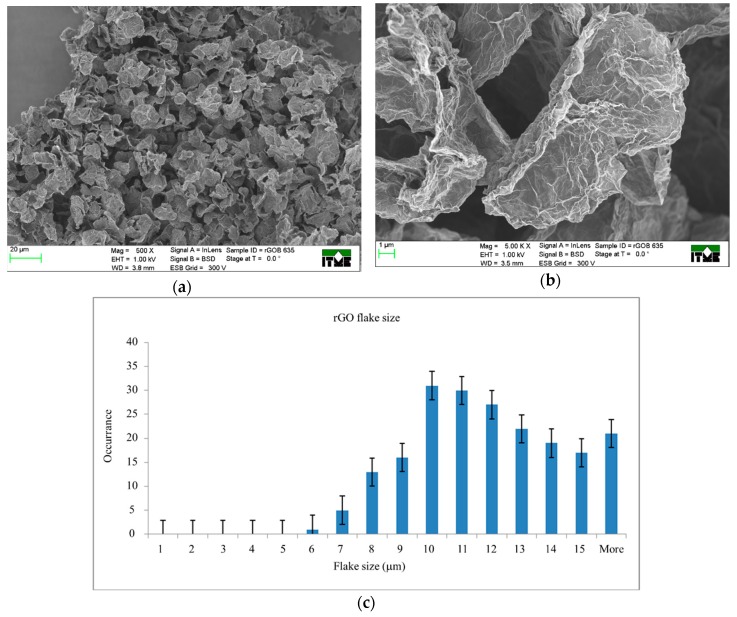
SEM images of reduced GO (rGO; B 635) at magnifications: (**a**) 500× and (**b**) 5.00 K × and (**c**) the size distributions of rGO flakes (population of over 200 flakes was taken under consideration).

The morphology of rGO was more uniform (Figure 2). The largest amount of flakes had a size of around 10 µm. The observed morphology was a result of the drying process, when the 3D structure consisting of flakes, was collapsing.

**Figure 3:**
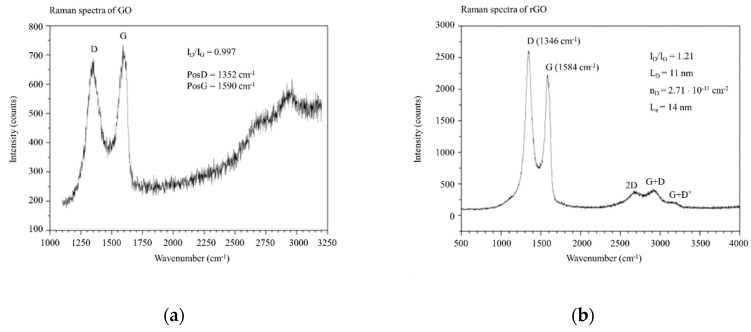
Raman spectra of (**a**) GO and (**b**) rGO flakes.

In the observed Raman spectra one could see two peaks, D and G. D peak gives information about defects in graphene, like carbon vacancies, 5-, 7- or 8-fold carbon rings or residues of oxygen groups, while G was a graphitic signature of the material (Figure 3). The ratio between the intensity of D and G peaks provided information about the order of the structure. For GO sample this ratio was about 0.99, which was much lower comparing to literature. Probably it was caused by gentle oxidation, which results in more saved sp^2^ domains. For the product of chemical reduction—rGO, this ratio was about 1.2. This means that more defects occurred or less sp^2^ areas were present. This could be explained by hard conditions of reducton of GO. In addition, the three peaks (2D, G+D and G+D’) appeared. Probably they were related to the restoration of the sp^2^ hybridization.

**Figure 4:**
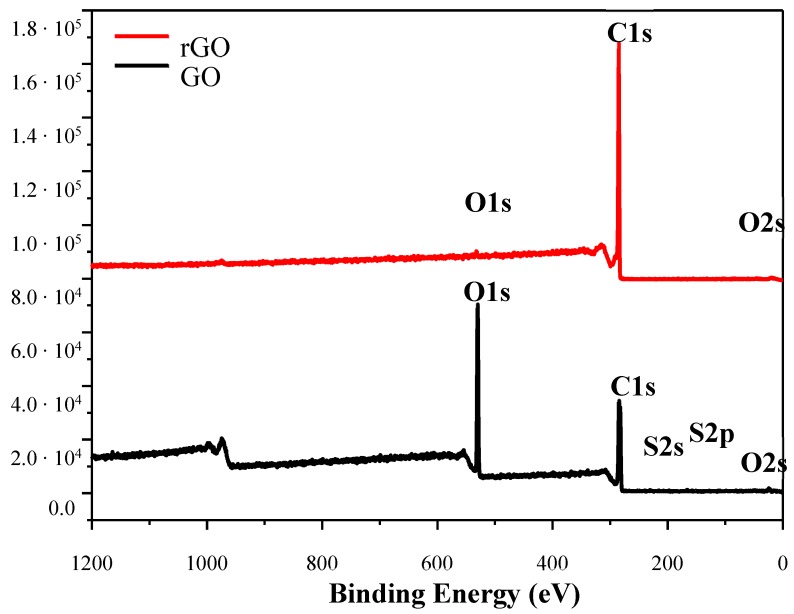
X-ray photoelectron wide range spectra of GO and rGO samples.

Wide range X-ray photoelectron spectra of GO and rGO (Figure 4) show the elemental composition of graphene materials. For GO a strong signal from the oxygen 1s orbital was visible, and also a weak signal from the 2s orbital was registered. The spectrum also contained a clear peak that originated from the carbon 1s orbital and weak signals from sulfur orbitals. For rGO sample, a strong signal from the 1s carbon orbital was observed. The residues of oxygen groups give signals from 1s and 2s orbitals. The changes in the recorded spectra of GO and rGO were the result of the reduction process.

The reduction process eliminates oxygen groups from the surface and edges of flakes. Using sodium hypophosphite as a reducing agent the oxygen content could be lowered to 15% by weight (Table 1).

**Figure 5:**
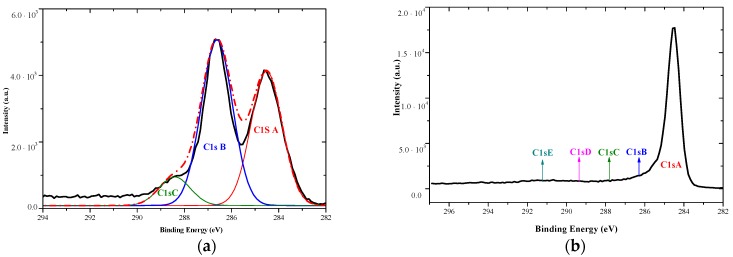
XPS spectra deconvolution of carbon peaks for (**a**) GO and (**b**) rGO samples.

Analysis of carbon peaks allowed for assigning deconvoluted curves to different types of bindings between carbon atoms and carbon and oxygen atoms (Figure 5). In the case of GO, aromatic bonds, hydroxyl, epoxy and phenolic groups were observed. The peaks intensity shows, what kinds of bonds constituted the majority of atomic bonds in the material. The deconvoluted curves of rGO reveal the presence of aromatic bonds, which were inherent in graphene nature. After the reduction process, some carboxylic, phenol, epoxy and ester groups were still present in the material, that was typical for products of chemical reduction of GO (Table 2).

The elemental analysis of GO and rGO samples confirmed the results obtained by the XPS (Table 3). The content of elements in both materials was in the typical range for these graphene derivatives. The source of sulfur was sulphuric acid used in the oxidation step. The decreased content of oxygen in rGO was caused by the reduction process, and the oxygen concentration indicates that the sodium hypophosphite was an efficient reducing agent for GO.

**Figure 6:**
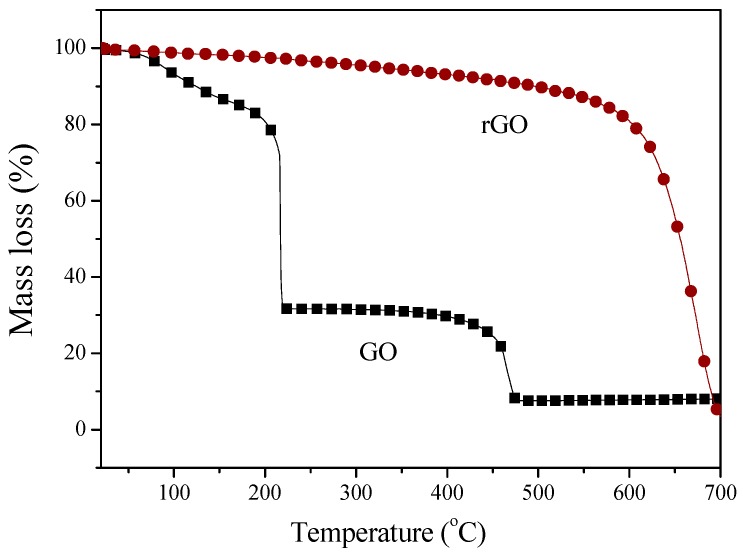
Thermogravimetric analysis of GO and rGO in an oxidizing atmosphere.

The TGA curves of the as-received GO and rGO nanoparticles depict continuous weight loss, as shown in Figure 6. The TGA curves for GO show two major mass losses between 100 and 700 °C. Nevertheless, a small mass loss of about 5% at around 100 °C could be assigned to the removal of water molecules trapped inside the GO structure. However, a first rapid mass loss at 220 °C corresponded to the removal of most of the oxygen-containing functional groups from the structure of GO (almost 68% of the starting mass) [34,35]. The second mass loss of about 24% at 480 °C could be ascribed to the thermal decomposition of GO. However, there was still ca. 7% of GO residue until the temperature of 700 °C. The above-presented results are easy to understand: The oxidation of graphite, which has a layered morphology, with oxygen-containing functionality, weakened the van der Waals forces between layers. This can disrupt the hexagonal carbon basal planes on the interior of multilayered stacks of graphene oxide, thus accelerating the process of losing the weight [36]. After the chemical reduction of GO, the thermal stability of rGO was almost as good as the pristine graphite. In the case of rGO, a decrease of ca. 15% was observed until the temperature of 560 °C, followed by a clear decomposition of the material. After chemical reduction, the functional groups were removed from the graphene surface leading to the improvement of thermal stability.

### 2.2. Characterization Methods

Raman spectra of GO and rGO were examined on a confocal Raman microscope, Reinshaw. All samples were deposited on silicon wafers in powder.

The SEM images of GO and rGO were performed by Zeiss Auriga and Hitachi SU8230. For the observation, an InLens detector was used (secondary electrons). In the case of GO, beam energy was set on 0.5 kV, for rGO it was 1 kV. A sample of GO was prepared by drop-casting of highly diluted suspension on a silicon substrate. For rGO imaging, a small quantity of powder was deposited on conductive tape. In turn, the dispersion of GO and rGO in the PTT-*block*-PCL-T matrix was evaluated by scanning electron microscopy (SEM, JEOL JSM 6100). The samples for SEM analysis were cryofractured in liquid nitrogen and subsequently coated (2–5 nm) in a vacuum with a thin gold film. 

XPS spectra were registered on the analytic system UHV Prevac. Each sample was placed on molybdenum carrier, and degassed in room temperature, down to ~5 × 10^−9^ mbar. The lamp VG Scienta Sax 100 with VG Scienta XM 780 monochromator was used as a source of x-rays. For registration of photoelectrons the hemisphere analyzer Scienta R4000 was used.

The basic physico-chemical properties of the polymer/nanocomposites samples were determined, by means of intrinsic viscosity, density, softening temperature and cold and boiling water absorption measurements. The intrinsic viscosity (η) of the samples was determined at the temperature of 30 °C in the mixture of phenol/1,1,2,2-tetrachloroethane (60/40 by weight) (Sigma-Aldrich). The polymer solution had a concentration of 0.5 g/dL. The measurement was carried on a capillary Ubbelohde viscometer (type Ic, K = 0.03294) following the procedure described in [33]. The measurements of the density of the dumbbell shape samples were carried out at 23 °C on hydrostatic scales (Radwag WPE 600C, Poland), calibrated according to standards with a known density. While the softening temperatures of the polymer/nanocomposites samples were determined using the Boethius apparatus. The method’s principle was to observe the moment when the edges of the samples start to melt as a result of constantly increasing temperature. In turn, water absorption tests were conducted in cold and boiling water in accordance with the test procedures recommended in ASTM D570, following the procedure described previously in [37].

The calorimetric measurements were carried out by means of differential scanning calorimetry (DSC) using a differential calorimeter Q-100 (TA Instruments, USA, 2004). All samples of about 10 mg were subjected to the cycle of heating–cooling–heating in the temperature range of 25 to 250 °C at the heating rate of 10 °C/min. The values of temperatures of physical transitions, i.e., glass-transition temperature (*T_g_*), crystallization temperature (*T_c_*) and melting temperature (*T_m_*) were designated. The *T_g_* was taken as the midpoint of the change in heat capacity. Additionally, the degree of crystallinity of nanocomposites was calculated using the following equation:

$$X_{c}\left(\%\right)=\frac{\Delta H_{m}}{\Delta H_{m}^{{}^{\circ}}},$$

where Δ$H_{m}^{{}^{\circ}}$ is the melting enthalpy of 100% crystalline sample, for PTT equals 146 J/g [38] and Δ*H_m_* is derived from melting peak area on DSC thermograms.

The thermal and thermo-oxidative stability measurements of as received GO, rGO and nanocomposites were carried out in a SETARAM TGA 92.16 thermogravimeter at a heating rate of 10 °C/min in argon and dry, synthetic air (N_2_:O_2_ = 80:20 vol.%) in the atmosphere, respectively. The study was conducted in the temperature range of 20–700 °C. Measurements were performed in accordance with the PN-EN ISO 11358:2004 standard.

Electrical conductivity measurements were performed at room temperature in the frequency range from 10^−2^ Hz to 106 Hz using a Novocontrol broadband dielectric spectrometer as previously described [39,40,41].

The thermal conductivity coefficient of the prepared materials was determined by the transient plane source (TPS) method using the Hot Disk TPS 2500 S (Uppsala, Sweden), and the Hot Disk thermal constants analyzer. The measurement was performed according to ISO 22007-2. The measurements were carried out on the middle part of the sample sheet with a penetration depth of 3.5–3.9 μm. All specimens with a thickness of approximately 2 mm were placed on both sides of the Hot Disk sensor touching the sensor with plane surfaces.

Hardness measurements were performed using the Shore D apparatus (Karl Frank GmbH, Type 104, Germany) according to a standard DIN 53505 and ISO 868.

The mechanical performance of the prepared materials was evaluated using Autograph AG-X plus (Shimadzu) tensile testing machine equipped with a 1 kN Shimadzu load cell, an optical extensometer, and the TRAPEZIUM X computer software, operated at a constant crosshead speed of 5 mm/min, following the procedure previously described in [33]. According to PN-EN ISO 527 standard, the tensile modulus, yield stress and strain, stress and elongation at break of the nanocomposites were determined. Five measurements were conducted for each sample, and the results were averaged to obtain a mean value.

### 2.3. Preparation Procedure

#### 2.3.1. Synthesis of Polymer Nanocomposites

The poly(trimethylene terephthalate)-*block*-polycaprolactone (PTT-*block*-PCL-T) copolymer with the content of 75 wt. % of PTT segments and 25 wt. % of PCL-T segments and nanocomposites were prepared by in situ polymerization in 1 dm^3^ steel high-pressure reactor (Autoclave Engineers Pennsylvania, USA), following the procedure described in our previous work [33]. Graphene oxide (GO) and reduced graphene oxide (rGO) were dispersed in 400 mL of bio-PDO using ultra-high speed stirrer (Ultra-Turax T25, Wilmington, NC, USA) for 15 min and afterwards stirred by ultrasonicator (Homogenizer HD 2200, Sonoplus, Berlin, Germany) with the frequency of 20 kHz and power of 200 W. Subsequently, the dispersion was subjected to ultrasounds in the ultra-power lower sonic bath (BANDELIN, Sonorex Digitec, Berlin, Germany) with frequency of 30 kHz and power 140 W for 15 min. The dispersion was added into the reaction mixture. The polymerization process was conducted in two steps: transesterification and polycondensation. Transesterification was carried out under a constant flow of nitrogen in the temperature range of 165–180 °C for about two hours. The second step (polycondensation) began when the amount of by-product (methanol) was about 90% of the stoichiometric calculated value. Then the thermal stabilizer and the second part of the catalyst were added into the reactor and the temperature was slowly increased to 245 °C and the pressure was reduced to 20 Pa. During the polycondensation, the stirring torque changes were monitored to evaluate the viscosity of the product. All syntheses were finished when melt reached the established value of viscosity at 245 °C. The molten nanocomposite was extruded into the water bath and then granulated and dried before processing.

#### 2.3.2. Samples Preparation

The dumbbell shaped samples (type A3) for tensile tests and thermal conductivity were obtained by injection molding using a Boy 15 (Dr BOY GmbH and Co., Germany) injection molding machine with the following parameters: injection pressure 80 MPa, melt temperature 225 °C, mold temperature 30 °C, holding down pressure of 20 MPa for 15 s and cooling time of 10 s. Samples for electrical conductivity study were prepared in the form of thin polymer foils with a thickness of ≈ 200 ± 5 μm by press molding (Collin P 200E) at 230 °C, and pressure of 5 bar for 2 min and 10 bar for another 1 min and subsequently quenched in cold water. The thickness of thin films was measured with a Micrometer mod. 293–521 from Mitutoyo. Five measurements were taken for each sample, with an experimental error of ±0.001 mm. The thickness is an average value.

## 3. Results and Discussion

### 3.1. Dispersion of Graphene Oxide and Reduced Graphene Oxide in the Polymer Matrix

**Figure 7:**
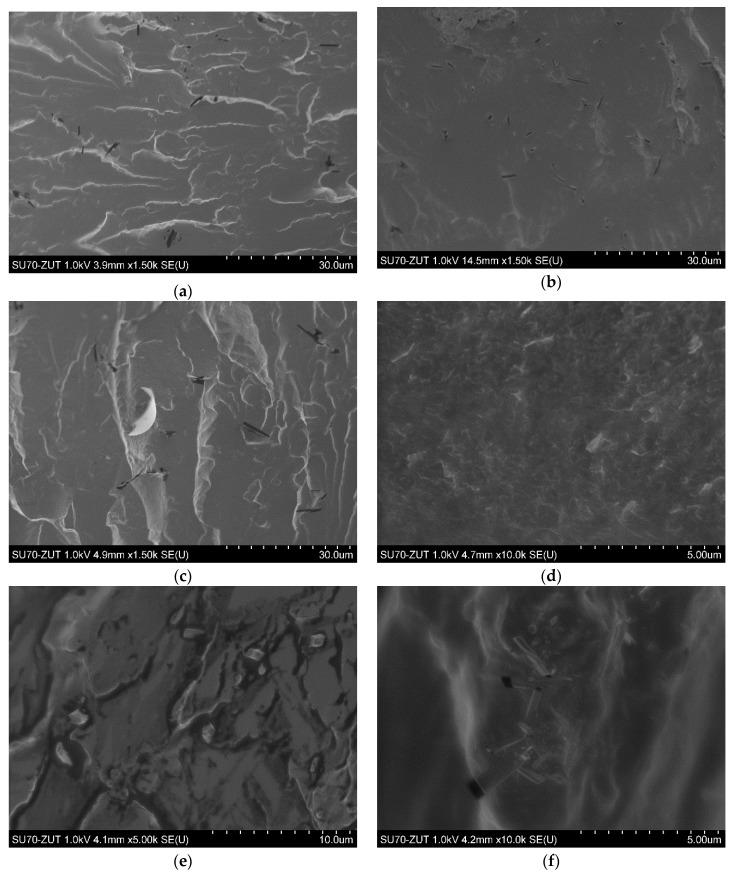
SEM micrographs of PTT-*block*-PCL-T nanocomposites containing: (**a**) 0.1 GO; (**b**) 0.1 rGO; (**c**) 0.5 GO; (**d**) 0.5 rGO; (**e**) 1.0 GO and (**f**) 1.0 rGO.

Morphological observations of PTT-*block*-PCL-T based nanocomposites were made using SEM. Figure 7a,c,e show SEM images of nanocomposites containing 0.1, 0.5 and 1.0 wt.% of GO, respectively, while Figure 7b,d,f show SEM images of nanocomposites containing 0.1, 0.5 and 1.0 wt. % of rGO, respectively. As one can observe, the high shear forces along with alternately applied ultrasounds introduced by high-speed mechanical stirring and ultrasonication were sufficient to disperse both GO and rGO uniformly in the PTT-*block*-PCL-T matrix. Despite the fact that it is extremely difficult to obtain a stable dispersion of nanoplatelets in the polymer matrix, due to strong intermolecular forces that exist between nanoparticles, in this case, it was possible to obtain well-dispersed graphene oxide and reduced graphene oxide, regardless the concentration of nanoplatelets in the polymer matrix.

### 3.2. Physico-Chemical Properties of PTT-Block-PCL-T Nanocomposites 

In order to verify the influence of the addition of GO and rGO on the synthesis process of PTT-*block*-PCL-T, intrinsic viscosity measurements along with density, softening temperature and cold and hot water absorption tests were performed (Table 4). As expected, the results show that the intrinsic viscosity of PTT-*block*-PCL-T decreased along with the increase in the concentration of both, GO and rGO. Especially, one can see a decrease of about 27% and 34% for the highest concentration (1 wt. %) of GO and rGO, respectively. In addition, as the GO and rGO loading level increased, the density of the obtained nanocomposites increased due to the presence of nanofiller with higher density. Similarly, along with the increase of GO and rGO concentration, an increase of softening temperature was observed (Boethius method). Such increase results from an influence of both GO and rGO on the molecular mobility of polymer chains, and thus the stiffening effect of the polymer matrix, which was further confirmed by DSC analysis and tensile tests.

Moreover, one can find that a small amount of GO and rGO could enhance the barrier properties of the block copolymer, which was observed as lowering the value of absorption of cold and boiled water. Here, the cold water absorption (CWA) and hot water absorption (HWA) represent the percentage of water absorbed by the nanocomposites within a certain time at a certain temperature (according to standards). The higher the absorption ratio, the better the permeability of water into the nanocomposites is, and the poorer the barrier properties of the final material are. In the present study one observed that the incorporation of GO and especially rGO reduces the water absorption. The CWA for neat PTT-*block*-PCL-T was 0.52%, while the value of HWA was 0.17%, thus suggesting that the permeability of water in the nanocomposites decreased modestly along with an increase in the number of nanoplatelets. The greatest impact was observed for the nanocomposites containing 1.0 wt. % of rGO (CWA increased by 16%). Nevertheless, also from the HWA measurements, one confirmed the promising behavior of graphene derivatives in improving the permeability of the materials. A more pronounced effect on improving barrier properties toward cold and boiling water was observed for rGO. It is due to the fact, that GO is hydrophilic, it contains oxygen groups, while rGO is hydrophobic as oxygen groups are removed in the reduction process. Therefore, the less oxygen-containing groups remaining on the graphene surface account for the better improvement in barrier properties toward the water, which is in the agreement with our previous study [2].

### 3.3. Thermal Properties (DSC and TGA)

**Figure 8:**
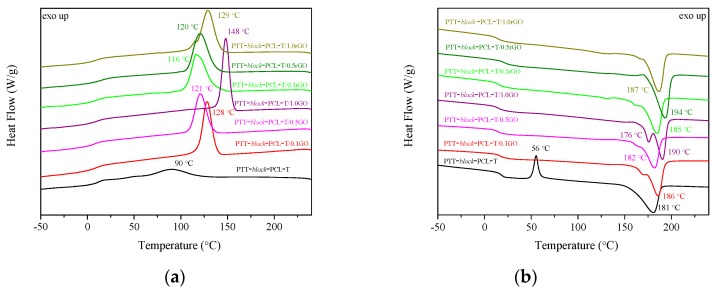
Differential scanning calorimetry (DSC) thermograms for neat PTT-*block*-PCL-T copolymer and PTT-*block*-PCL-T/GO, PTT-*block*-PCL-T/rGO composites recorded during (**a**) cooling and (**b**) second heating.

The DSC thermograms of PTT-*block*-PCL-T copolymer and PTT-*block*-PCL-T-based nanocomposites with GO and rGO are presented in Figure 8 and the results are summarized in Table 5. The glass transition temperature (*T_g_*) of neat PTT-*block*-PCL-T copolymer was 17 °C, melting point (*T_m_*) was around 181 °C and crystallization temperature T_c_ was 90 °C. Moreover, the PTT-*block*-PCL-T copolymer exhibited a cold crystallization peak at the temperature of 56 °C. A more detailed analysis of the whole series of block copolymers based on PTT (as rigid segment) and PCL-T (as flexible segment) was published in our previous work [33]. The use of GO and rGO did not cause significant changes in *T_g_*, but it slightly increased *T_m_*. The highest values of *T_g_* and *T_m_* were observed for PTT-*block*-PCL-T/0.5rGO composite. Moreover PTT-*block*-PCL-T/1.0GO composite exhibited two distinct T_m_ peaks at 176 °C and 190 °C. DSC curves of composites with 0.1 and 0.5 wt.% of GO contain less pronounced peaks of the second melting point, which suggests that the amount of GO filler influences the formation of PTT-*block*-PCL-T/GO crystals. Xing et al. [42] studied the PET/GO and PET/GL-g-LMPET nanocomposites and they observed double melting peaks after addition of GL-g-LMPET into the PET matrix. The lower temperatures were similar to the *T_m_* of neat PET, so they assumed that the lower *T_m_* was caused by the crystals of PET and the higher by crystals of PET/GL-g-LMPET. The crystallization temperature, that refers to the formation of crystals, increased with the addition of fillers from 90 °C (neat PTT-*block*-PCL-T copolymer) to 148 °C (PTT-*block*-PCL-T/1.0GO composite). Addition of GO and rGO to PTT-*block*-PCL-T copolymer caused a significant increase in enthalpy of crystallization *ΔH_c_*, enthalpy of melting *ΔH_m_* and the degree of crystallinity. Furthermore, the incorporation of GO affected the crystallization behavior stronger than rGO, thus increasing the degree of crystallinity of about 20% (PTT-*block*-PCL-T/1.0GO). Nogueira de Melo et al. [43] also observed that the presence of GO in polyamine 6 (PA6) affected the enthalpies of crystallization and melting and in consequence, it enhanced the degree of crystallinity. Moreover, the values of heat capacity were comparable to one another in the whole series of materials regardless of the incorporation of GO and rGO.

**Figure 9:**
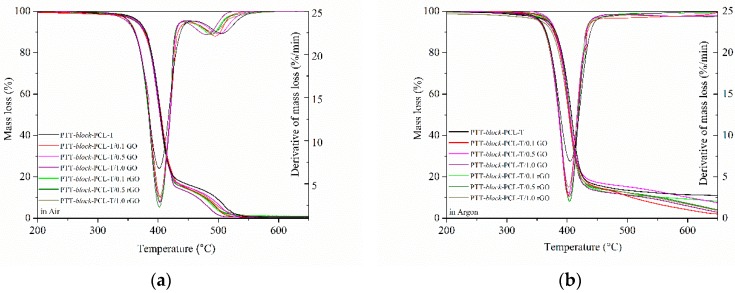
Mass loss and derivative of mass loss as a function of temperature for PTT-*block*-PCL-T based nanocomposites containing GO and rGO in (**a**) oxidizing (air) and (**b**) in an inert atmosphere (argon).

The thermo-oxidative and thermal stability of neat PTT-*block*-PCL-T and the composites with GO and rGO were investigated using thermogravimetric analysis (TGA). The mass loss (TG) and derivative of mass loss (DTG) curves are presented in Figure 9. The characteristic temperatures of 5%, 10% and 50% of mass loss (in an oxidizing and inert atmosphere) are summarized in Table 6. In an oxidizing atmosphere the neat copolymer and the series of composites exhibited two stages of degradation in the temperature range of 320–440 °C and 450–540 °C. The first stage of degradation is related to the decomposition of flexible and rigid segments and the second one is related to the decomposition of the residue. The temperatures of 5% and 10% of mass loss in an oxidizing atmosphere were slightly lower for composites containing 0.1 wt.% and 1.0 wt.% of GO compared with the neat PTT-*block*-PCL-T. Bai et al [44] had the same situation with GO/SR nanocomposites. They observed that the decomposition temperature in thermo-oxidative stability decreased after the addition of GO filler. They indicated that it might be caused by the presence of oxygen groups, which might accelerate the oxidization of side groups and this could play a negative role in the thermal oxidative stability. The temperatures of 5% and 10% mass loss for composite with 0.1 wt.% of rGO were also lower of about 2 °C than for neat PTT-*block*-PCL-T. The temperatures of 50% mass loss for all composites were similar to those obtained for neat PTT-*block*-PCL-T. No significant changes in the first stage of derivative mass loss temperatures (T_DTG1_) were observed, however, some changes in the temperatures in the second stage (T_DTG2_) were noticed. Temperatures of obtained composites were lower than for the neat PTT-*block*-PCL-T, they shifted from 506 °C to 479 °C (1.0 wt.% of GO) and 488 °C (0.1 wt.% of rGO). 

Measurements in an inert atmosphere show only one stage of the degradation process in the temperature range of 325–460 °C. The temperatures of 5% of mass loss increased with increasing content of fillers and the highest temperatures reached composites with 0.5 wt.% content of fillers. Further increase in the filler content caused a decrease in these temperatures for composites. In turn, the temperatures of 10% and 50% of mass loss slightly decreased with increasing content of fillers. Temperatures of derivative mass loss (T_DTG1_) of composites were similar to the temperature obtained for neat PTT-*block*-PCL-T. Moreover, the values of activation energies (E_a_) for the PTT-*block*-PCL-T-based nanocomposites increased with increasing content of GO and rGO, when the measurement was carried out in an oxidizing and in an inert atmosphere (Table 6). The values of E_a_ for the measurement carried out in air ranged between 81–86 kJ/mol, whereas the highest value was observed in the case of nanocomposites containing 0.5 wt.% of nanofiller, which probably results from the quality of the dispersion of the sample. In turn, for the measurement carried out in argon these values were in the range 77–82 kJ/mol. The differences in the values of E_a_ for PTT-*block*-PCL-T and nanocomposites suggest that polymer-GO or polymer-rGO interactions support the rupturing of polymer bonds in oxidizing and pyrolysis conditions.

### 3.4. The Thermal and Electrical Conductivity of the Samples

**Figure 10:**
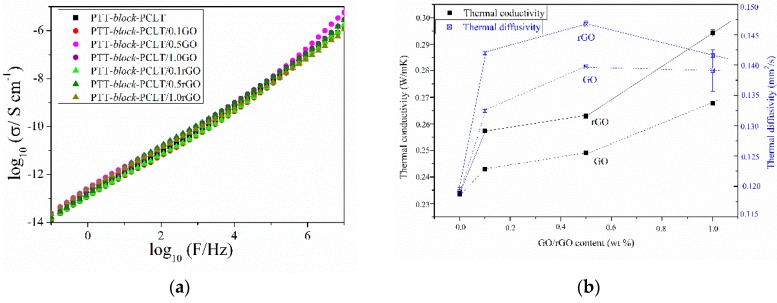
(**a**) Broadband electrical conductivity as a function of frequency at room temperature for neat PTT-*block*-PCL-T and PTT-*block*-PCL-T-based nanocomposites and (**b**) thermal conductivity and thermal diffusivity vs. nanofillers’ content for neat PTT-*block*-PCL-T and PTT-*block*-PCL-T-based nanocomposites.

The remarkable electrical conductivity of graphene and its derivatives, like few layers of graphene, graphene nanoplatelets or even expanded graphite can be utilized to transform traditionally insulating polymer matrices into electrically conductive materials for various applications such as conductive adhesives, antistatic coatings and films [2,40,45]. There is a critical concentration of fillers (percolation threshold—“PT”) at which the composite transits from an insulator to a conductor due to the formation of a continuous conducting network: i) below the PT, the electrical properties are dominated by the dielectric properties of polymer matrix and thus the composite is non-conductive and ii) above the PT, a small increase in concentration results in a significant increase in conductivity (the nanoparticles begin to form a contact with each other). Electrical conductivity and percolation threshold in a polymer nanocomposite is related to the inherent conductivity of the nanoparticle, its concentration, aspect ratio, extent of aggregation and dispersion, orientation and finally directed assembly of the nanoparticles in the polymer matrix [46]. Unfortunately, in both series of PTT-*block*-PCL-T- based nanocomposites containing GO and rGO even at the loading of 1.0 wt.% no improvement in electrical conductivity was observed (Figure 10a). All synthesized materials exhibited a typical insulating behavior, and since the dispersion of nanofillers in the whole volume of polymer matrix was rather homogenous, this probably results from several factors: i) the loading of 1.0 wt.% was not high enough to create the percolation paths in the multiphase copolymer system [47]; ii) a higher degree of crystallinity of nanocomposites in comparison to neat polymer matrix, which along with the differences in the anisotropy of the materials can affect the orientation of nanoplatelets, while percolating behaviors are observed only when particles are aligned parallel [40] and iii) the quality of GO and rGO, from the Raman spectroscopy and XPS, one can find that the structure of both nanofillers was rather full of irregularities and defects (ID/IG ≈ 1) and contained ca. 50% of oxygen, in the case of GO, and about 15%–20% in the case of rGO, which confirms that the chemical reduction had not restored the electrical conductivity of GO [19,47,48]. 

Along with electrical conductivity thermal conductivity and the thermal conductivity and thermal diffusivity of PTT-*block*-PCL-T-based nanocomposites containing GO or rGO were also studied (Figure 10b). It was found that the incorporation of both nanofillers caused almost linear enhancement in thermal conductivity of polymer matrix, while the highest value of thermal diffusivity was observed for the nanocomposites containing 0.5 wt.% of rGO. The more pronounced increase observed in the case of rGO was probably due to two main reasons: Firstly, the presence and features of a crystalline structure (PTT), which affects heat transfer in both the polymer phase at the interface between nanoplatelets and polymer [49] and, secondly, the homogeneous dispersion, confirmed by SEM observations, that lets creating an interconnecting network suitable for heat transfer. Such an enhancement in thermal conductivity (linearly increasing with increasing content of nanoplatelets) is in agreement with our previous observations made on PTT-PTMO/SWCNTs+GNP hybrid nanocomposites prepared by in situ polymerization [50].

### 3.5. Tensile Properties of PTT-block-PCL-T Nanocomposites 

**Figure 11:**
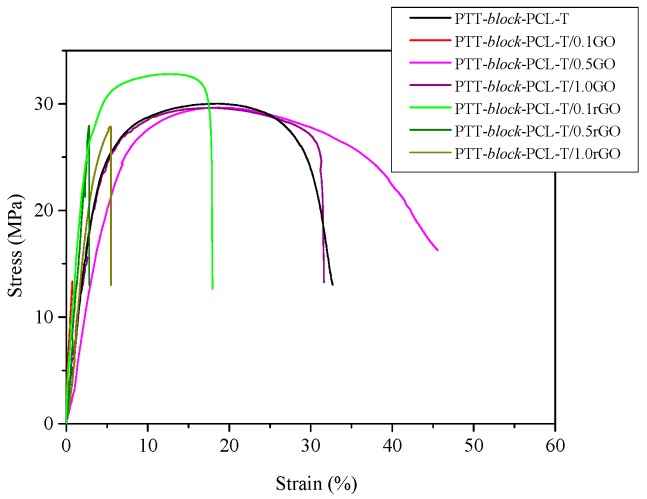
Representative stress–strain curves for both series of PTT-*block*-PCL-T based nanocomposites containing GO and rGO.

The mechanical properties of the synthesized composites are expected to get improved with the addition of GO and rGO. Graphene oxide is a good strengthening filler because of its high intrinsic mechanical properties and good load transfer from the matrix to the reinforcing phase [20,21,22]. rGO also has good mechanical properties such as elastic modulus, which is similar to the value predicted for pristine graphene [28]. The mechanical properties of the composites were evaluated by shore hardness and tensile tests. The obtained representative stress–strain curves of composites are presented in Figure 11. The characteristic parameters such as shore hardness, Young’s modulus (E), maximum tensile strength (σ_M_), tensile strength and elongation at break (σ_b_, ε_b_) are summarized in Table 7. The prepared composites reveal an increase in hardness along with increasing the content of nanofillers. The highest value was reached by the composite with 1.0 wt.% of GO (~66 Sh D) and it was about 10% higher than obtained for neat PTT-*block*-PCL-T. 

Along with an increase of fillers content, the increase in the values of Young’s modulus, maximum tensile strength and tensile strength at break were observed. It was related to the good mechanical properties of graphene oxide and reduced graphene oxide. Young’s modulus value increased over three times for both composites containing 0.1 wt.% of fillers, but further addition of them caused the decrease of this value. 

The addition of 1.0 wt.% GO caused enhancement of maximum tensile strength (σ_M_) and strength at break (σ_b_) of about 1.61 MPa and 1.02 MPa, respectively if compared to the neat PTT-*block*-PCL-T. For rGO composites the highest value of σ_M_ and σ_b_ was reached with the lowest loading of rGO. The value of maximum tensile strength increased from 29.65 MPa (for PTT-*block*-PCL-T) to 32.89 MPa (for PTT-*block*-PCL-T/0.1 rGO) and strength at break increased from 26.69 MPa (for PTT-*block*-PCL-T) to 29.58 MPa (for PTT-*block*-PCL-T/0.1 rGO). The elongation at break values were higher for composites containing GO as a filler than for those with rGO and the highest value was reached by the composite with 0.5 wt.% of GO (33.13%).

## 4. Conclusions

Two series of nanocomposites based on PTT-*block*-PCL-T were synthesized by in situ polymerization and the effect of GO and rGO on the morphology and functional properties of polymer matrix were studied. One found that the utilized polymerization process allowed us to obtain homogeneously distributed nanoplatelets in the whole volume of the polymer matrix, which resulted in an improvement of mechanical properties. The interactions between the polymer matrix and the functional groups on the surface of GO and residual functional groups on the surface of rGO caused the enhancement in barrier properties (lower values of cold and boiling water absorption), thermal stability and thermal conductivity. It was also reported that GO and rGO were acted as nucleating agents and affect polymer crystallinity. Only a small effect on the molecular mobility was visible, which was concluded from the observations of glass transition and an increase of the value of softening temperature. Since, the electrical conductivity of the final material depends on the method of synthesis of nanofiller, its surface modification and particularly on the number of defects (reactive sites) generated during the oxidation–reduction process on the surface, one could not observe the improvement in electrical conductivity for any series of nanocomposites. Nevertheless, nanocomposites based on both GO or rGO might find some applications in advanced composites, replacing carbon fibers, or even in the energy conversion/storage devices or thermal management. 

## Figures and Tables

**Table 1:** The elemental compositions of GO and rGO samples calculated from the XPS spectra.

GO	Peak BE (eV)	Concentration at. (%)	Concentration Mass (%)	rGO	Peak BE (eV)	Concentration at. (%)	Concentration Mass (%)
C 1s	284.5	71.4	64.9	C 1s	284.5	89.2	85.8
O 1s	530.5	28.1	34	O 1s	532	10.5	13.5
S 2p	166	0.5	1.2	N 1s	399.5	0.3	0.7

**Table 2:** Bond contents present in GO and rGO materials calculated from XPS deconvoluted spectra.

Bond Type	GO	Peak BE (eV)	Concentration at. (%)	rGO	Peak BE (eV)	Concentration at. (%)
C=C sp^2^	C 1s A	284.5	46.1	C 1s A	284.5	77
C–O–C, C–OH	C 1s B	286.6	47.7	C 1s B	286.2	9.2
C=O				C 1s C	287.4	5.2
O=C–O–	C 1s C	288.4	6.2	C 1s D	289.4	4.2
π–π *				C 1s E	290.7	4.4

**Table 3:** Elemental analysis of GO and rGO.

Element	GO	rGO
Carbon	40%–42%	80%–85%
Oxygen	49%–52%	15%–18%
Sulphur	1%–3%	<2%
Nitrogen	<0.3%	<0.3%
Hydrogen	2.5%–3%	<2%

**Table 4:** Basic physico-chemical properties of PTT-*block*-PCL-T- based nanocomposites.

Sample	*η* (dl/g)	*d* (g/cm^3^)	*T_B_* (°C)	CWA (%)	HWA (%)
PTT*-block*-PCL-T	0.864	1.2932	195	0.52	0.17
PTT*-block*-PCL-T/0.1GO	0.851	1.2964	198	0.50	0.15
PTT*-block*-PCL-T/0.5GO	0.848	1.2941	196	0.50	0.14
PTT*-block*-PCL-T/1.0GO	0.636	1.3042	201	0.52	0.14
PTT*-block*-PCL-T/0.1rGO	0.785	1.2939	199	0.51	0.16
PTT*-block*-PCL-T/0.5rGO	0.729	1.3061	205	0.46	0.14
PTT*-block*-PCL-T/1.0rGO	0.559	1.3027	201	0.44	0.13

*η*—intrinsic viscosity; *d*—density; *T_B_*—softening temperature tested according to Boethius method; CWA—cold water absorption and HWA—hot water absorption.

**Table 5:** Thermal properties determined from cooling and 2nd heating thermograms for PTT and PTT-*block*-PCLT copolymers.

Sample	*T_g_* (°C)	Δ*C_p_* (J/g∙°C)	*T_c_* (°C)	Δ*H_c_* (J/g)	*T_m_* (°C)	Δ*H_m_* (J/g)	*X_c_* (%)
PTT*-block*-PCL-T*	17	0.22	90	20	181	29.5	14.4
PTT*-block*-PCL-T/0.1GO	18	0.24	128	40.5	186	41.9	28.7
PTT*-block*-PCL-T/0.5GO	15	0.21	121	40.1	182	41.8	28.6
PTT*-block*-PCL-T/1.0GO	16	0.21	148	49.5	176/190	50.6	34.6
PTT*-block*-PCL-T/0.1rGO	16	0.21	116	39.8	185	35.1	24.0
PTT*-block*-PCL-T/0.5rGO	21	0.25	120	42.6	194	45.3	31.0
PTT*-block*-PCL-T/1.0rGO	15	0.21	129	47.4	187	46.6	31.9

*: in this case the cold crystallization appears, with *T_CC_* = 56 °C, Δ*Hcc* = 8.4 J/g [29]; *T_g_*—glass transition temperature; Δ*C_p_*—change of heat capacity; *T_c_*, Δ*H_c_*—crystallization temperature and corresponding enthalpy of crystallization; *T_m_*, *ΔH_m_*—melting temperature and corresponding enthalpy of melting and *X_c_*—degree of crystallinity.

**Table 6:** TGA data: Temperatures of 5%, 10% and 50% mass loss, the temperatures corresponding to the maximum of mass losses (T_DTG1_ and T_DTG2_), activation energies (*E_a_*) and correlation coefficient in linear regression (R) in an oxidizing and an inert atmosphere.

Sample	T_5%_(°C)	T_10%_ (°C)	T_50%_ (°C)	T_DTG1_(°C)	*E_a_*, (R) (kJ/mol)	T_DTG2_ (°C)
Measurement in an oxidizing atmosphere						
PTT-*block*-PCL-T	367	379	404	402	81.97 (0.9983)	506
PTT*-block*-PCL-T/0.1GO	363	376	405	404	81.62 (0.9988)	495
PTT*-block*-PCL-T/0.5GO	367	378	404	403	86.65 (0.9990)	502
PTT*-block*-PCL-T/1.0GO	363	378	403	403	83.18 (0.9979)	479
PTT*-block*-PCL-T/0.1rGO	365	377	404	404	82.29 (0.9985)	488
PTT*-block*-PCL-T/0.5rGO	368	380	403	402	86.53 (0.9990)	491
PTT*-block*-PCL-T/1.0rGO	367	380	403	402	81.85 (0.9981)	491
Measurement in an inert atmosphere						
PTT-*block*-PCL-T	367	381	407	405	77.38 (0.9999)	-
PTT*-block*-PCL-T/0.1GO	369	379	403	402	82.96 (0.9999)	-
PTT*-block*-PCL-T/0.5GO	381	373	404	403	78.49 (0.9999)	-
PTT*-block*-PCL-T/1.0GO	365	377	404	404	79.10 (1.0000)	-
PTT*-block*-PCL-T/0.1rGO	369	379	404	404	79.99 (0.9998)	-
PTT*-block*-PCL-T/0.5rGO	372	381	405	404	79.10 (0.9999)	-
PTT*-block*-PCL-T/1.0rGO	359	375	403	403	80.23 (0.9998)	-

*E_a_*—energy activation; (R)—correlation coefficient in linear regression.

**Table 7:** Tensile properties of PTT-*block*-PCL-T copolymers.

Sample	Hardness (Sh D)	*E* (MPa)	σ_m_ (MPa)	σ_b_ (MPa)	*ε_b_* (%)
PTT*-block*-PCL-T	59 ± 3	528.54 ± 36.78	29.65 ± 0.43	26.69 ± 0.39	26.91 ± 2.92
PTT*-block*-PCL-T/0.1GO	60 ± 4	1633.67 ± 152.43	11.19 ± 1.47	7.49 ± 0.65	0.71 ± 0.04
PTT*-block*-PCL-T/0.5GO	64 ± 6	601.57 ± 65.21	29.32 ± 0.37	26.67 ± 0.33	33.13 ± 2.05
PTT*-block*-PCL-T/1.0GO	66 ± 2	419.96 ± 33.52	31.26 ± 2.46	27.71 ± 2.51	30.87 ± 0.66
PTT*-block*-PCL-T/0.1rGO	62 ± 3	1650.19 ± 122.34	32.89 ± 0.95	29.58 ± 0.85	16.99 ± 2.44
PTT*-block*-PCL-T/0.5rGO	61 ± 3	915.03 ± 45.73	26.98 ± 4.32	21.12 ± 2.51	2.66 ± 0.49
PTT*-block*-PCL-T/1.0rGO	62 ± 6	434.18 ± 23.78	27.19 ± 3.98	20,17 ± 0.48	5.13 ± 0.73

E—Young’s modulus (calculated from strain 0.05% to 0.25%); σ_M_—max tensile strength, σ_b_, *ε_b_*—strength and elongation at break, respectively.

## References

[1] Henning Storz K.-D.V. (2013). Bio-based plastics: Status, challenges, and trends. Appl. Agric. For. Res..

[2] Paszkiewicz S., Janowska I., Pawlikowska D., Szymczyk A., Irska I., Lisiecki S., Stanik R., Gude M., Piesowicz E. (2018). New functional nanocomposites based on poly(Trimethylene 2,5-furanoate) and few layer graphene prepared by in situ polymerization. Express Polym. Lett..

[3] Muñoz-Guerra S., Lavilla C., Japu C., Martínez De Ilarduya A. (2014). Renewable terephthalate polyesters from carbohydrate-based bicyclic monomers. Green Chem..

[4] Li S., Ernst Worrell M.P. (2010). Present and future development in plastics from biomass. Biofuels Bioprod. Biorefining.

[5] Chivrac F., Pollet E., Avérous L. (2009). Progress in nano-biocomposites based on polysaccharides and nanoclays. Mater. Sci. Eng. R Rep..

[6] Bordes P., Pollet E., Avérous L. (2009). Nano-biocomposites: Biodegradable polyester/nanoclay systems. Prog. Polym. Sci..

[7] Klemm D., Heublein B., Fink H.P., Bohn A. (2005). Cellulose: Fascinating biopolymer and sustainable raw material. Angew. Chemie Int. Ed..

[8] Huang X., Qi X., Boey F., Zhang H. (2012). Graphene-based composites. Chem. Soc. Rev..

[9] Mittal V., Kim S., Neuhofer S., Paulik C. (2016). Polyethylene/graphene nanocomposites: Effect of molecular weight on mechanical, thermal, rheological and morphological properties. Colloid Polym. Sci..

[10] Stankovich S., Dikin D.A., Dommett G.H.B., Kohlhaas K.A., Zimmney E.J., Stach E.A., Piner R.D., Nguyen S.B.T., Ruoff R.S. (2006). Graphene-based composite materials. Nature.

[11] Obrzut J., Migler K.B. (2009). Optical and Conductivity Properties from Liquid Phase Exfoliation of Natural Graphene. Graphene and Emerging Materials for Post-CMOS Applications.

[12] Chee W.K., Lim H.N., Huang N.M., Harrison I. (2015). Nanocomposites of graphene/polymers: A review. RSC Adv..

[13] Gao W., Gao W. (2015). Graphene Oxide Reduction Recipes, Spectroscopy, and Applications.

[14] Brodie B.C. (1960). Sur le poids atomique du graphite. Ann. Chim. Phys..

[15] Staudenmaier L. (1899). Verfahren zur Darstellung der Graphitsäure. Ber. Dtsch. Chem. Ges..

[16] Hummers W.S., Offeman R.E. (1958). Preparation of Graphitic Oxide. J. Am. Chem. Soc..

[17] Marcano D.C., Kosynkin D.V., Berlin J.M., Sinitskii A., Sun Z., Slesarev A., Alemany L.B., Lu W., Tour J.M. (2010). Improved synthesis of graphene oxide. ACS Nano.

[18] Loh K.P., Bao Q., Eda G., Chhowalla M. (2010). Graphene oxide as a chemically tunable platform for optical applications. Nat. Chem..

[19] Stankovich S., Dikin D.A., Piner R.D., Kohlhaas K.A., Kleinhammes A., Jia Y., Wu Y., Nguyen S.T., Ruoff R.S. (2007). Synthesis of graphene-based nanosheets via chemical reduction of exfoliated graphite oxide. Carbon N. Y..

[20] Yoo B.M., Shin H.J., Yoon H.W., Park H.B. (2014). Graphene and graphene oxide and their uses in barrier polymers. J. Appl. Polym. Sci..

[21] Gómez-Navarro C., Burghard M., Kern K. (2008). Elastic properties of chemically derived single graphene sheets. Nano Lett..

[22] Suk J.W., Piner R.D., An J., Ruoff R.S. (2010). Mechanical properties of monolayer graphene oxide. ACS Nano.

[23] Chen W., Yan L., Bangal P.R. (2010). Preparation of graphene by the rapid and mild thermal reduction of graphene oxide induced by microwaves. Carbon N. Y..

[24] Ping J., Wang Y., Fan K., Wu J., Ying Y. (2011). Direct electrochemical reduction of graphene oxide on ionic liquid doped screen-printed electrode and its electrochemical biosensing application. Biosens. Bioelectron..

[25] Shao Y., Wang J., Engelhard M., Wang C., Lin Y. (2010). Facile and controllable electrochemical reduction of graphene oxide and its applications. J. Mater. Chem..

[26] Chua C.K., Pumera M. (2014). Chemical reduction of graphene oxide: A synthetic chemistry viewpoint. Chem. Soc. Rev..

[27] Gómez-Navarro C., Weitz R.T., Bittner A.M., Scolari M., Mews A., Burghard M., Kern K. (2007). Electronic transport properties of individual chemically reduced graphene oxide sheets. Nano Lett..

[28] Moon I.K., Lee J., Ruoff R.S., Lee H. (2010). Reduced graphene oxide by chemical graphitization. Nat. Commun..

[29] Ramanathan T., Abdala A.A., Stankovich S., Dikin D.A., Herrera-Alonso M., Piner R.D., Adamson D.H., Schniepp H.C., Chen X., Ruoff R.S. (2008). Functionalized graphene sheets for polymer nanocomposites. Nat. Nanotechnol..

[30] Wang H., Wang Z., Wang X., Wang L., Cai Y., Hong K., Sun L., Lu G., Zhao D., Li Z. (2015). Styrene–butadiene–styrene copolymer-compatibilized interfacial-modified graphene oxide with mechanical and electrical properties. J. Therm. Compos. Mater..

[31] Paszkiewicz S., Szymczyk A., Spitalsky Z., Mosnacek J., Kwiatkowski K., Roslaniec Z. (2014). Structure and properties of nanocomposites based on PTT–block-PTMO copolymer and graphene oxide by in situ polymerization. Europ. Polym. J..

[32] Paszkiewicz S., Pawelec I., Szymczyk A., Roslaniec Z. (2015). Thermoplastic elastomers containing 2D nanofillers: Montmorillonite, graphene nanoplatelets and oxidized graphene platelets. Pol. J. Chem. Technol..

[33] Paszkiewicz S., Szymczyk A., Irska I., Pawlikowska D., Piesowicz E. (2019). Synthesis, structure and physical properties of poly(trimethylene terephthalate)-block-poly(caprolactone) copolymers. J. Appl. Polym. Sci..

[34] Shen J., Hu Y., Shi M., Lu X., Qin C., Li C., Ye M. (2009). Fast and facile preparation of graphene oxide and reduced graphene oxide nanoplatelets. Chem. Mater..

[35] Zhang Y., Ma H.L., Zhang Q., Peng J., Li J., Zhai M., Yu Z.Z. (2012). Facile synthesis of well-dispersed graphene by γ-ray induced reduction of graphene oxide. J. Mater. Chem..

[36] Yoo E., Kim J., Hosono E., Zhou H., Kudo T., Honma I. (2008). Large Reversible Li Storage of Graphene Nanosheet Families for Use in Rechargeable Lithium Ion Batteries. Nano Lett..

[37] Paszkiewicz S., Kwiatkowska M., Roslaniec Z., Szymczyk A., Jotko M., Lisiecki S. (2016). The influence of different shaped nanofillers (1D, 2D) on barrier and mechanical properties of polymer hybrid nanocomposites based on PET prepared by in situ polymerization. Polym. Compos..

[38] Pyda M., Boller A., Grebowicz J., Chuah H., Lebedev B.V., Wunderlich B. (1998). Heat Capacity of Poly (trimethylene terephthalate). J. Polym. Sci. Part B Polym. Phys..

[39] Szymczyk A., Roslaniec Z., Zenker M., Rueda D.R., Nogales A., Ezquerra T.A. (2011). Preparation and characterization of nanocomposites based on COOH functionalized multi-walled carbon nanotubes and on poly ( trimethylene terephthalate ). Express Polym. Lett..

[40] Paszkiewicz S., Szymczyk A., Špitalský Z., Soccio M., Mosnáček J., Ezquerra T.A., Rosłaniec Z. (2012). Electrical conductivity of poly(ethylene terephthalate)/expanded graphite nanocomposites prepared by in situ polymerization. J. Polym. Sci. Part B Polym. Phys..

[41] Paszkiewicz S., Szymczyk A., Janowska I., Jedrzejewski R., Linares A., Ezquerra T.A., Wagner H.D., Tenne R., Rosłaniec Z. (2017). Comparative study on the properties of poly(trimethylene terephthalate) -based nanocomposites containing multi-walled carbon (MWCNT) and tungsten disulfide (INT-WS2) nanotubes. Polym. Adv. Technol..

[42] Xing L., Wang Y., Wang S., Zhang Y., Mao S., Wang G., Liu J., Huang L., Li H., Belfiore L. (2018). Effects of Modified Graphene Oxide on Thermal and Crystallization Properties of PET. Polymers.

[43] de Melo C.C.N., Beatrice C.A.G., Pessan L.A., de Oliveira A.D., Machado F.M. (2018). Analysis of nonisothermal crystallization kinetics of graphene oxide—Reinforced polyamide 6 nanocomposites. Thermochim. Acta.

[44] Bai Y., Cai H., Qiu X., Fang X., Zheng J. (2015). Effects of graphene reduction degree on thermal oxidative stability of reduced graphene oxide/silicone rubber nanocomposites. High Perform. Polym..

[45] Paszkiewicz S., Szymczyk A., Pawlikowska D., Subocz J., Zenker M., Masztak R. (2018). Electrically and thermally conductive low density polyethylene-based nanocomposites reinforced by MWCNT or hybrid MWCNT/graphene nanoplatelets with improved thermo-oxidative stability. Nanomaterials.

[46] Paszkiewicz S., Szymczyk A. (2019). Graphene-Based Nanomaterials and Their Polymer Nanocomposites. Nanomaterials and Polymer Nanocomposites.

[47] Paszkiewicz S., Szymczyk A., Sui X.M., Wagner H.D., Linares A., Ezquerra T.A., Rosłaniec Z. (2015). Synergetic effect of single-walled carbon nanotubes (SWCNT) and graphene nanoplatelets (GNP) in electrically conductive PTT-block-PTMO hybrid nanocomposites prepared by in situ polymerization. Compos. Sci. Technol..

[48] Dresselhaus M.S., Jorio A., Hofmann M., Dresselhaus G., Saito R. (2010). Perspectives on carbon nanotubes and graphene. Raman spectroscopy. Nano Lett..

[49] Wang Y., Shan J.W., Weng G.J. (2015). Percolation threshold and electrical conductivity of graphene-based nanocomposites with filler agglomeration and interfacial tunneling. J. Appl. Phys..

[50] Paszkiewicz S., Szymczyk A., Pilawka R., Przybyszewski B., Czulak A., RosŁaniec Z. (2017). Improved Thermal Conductivity of Poly(trimethylene terephthalate-block-poly(tetramethylene oxide) Based Nanocomposites Containing Hybrid Single-Walled Carbon Nanotubes/Graphene Nanoplatelets Fillers. Adv. Polym. Technol..

